# Supplementary material for: Mechanisms governing the pioneering and redistribution capabilities of the non-classical pioneer PU.1
Source: Nat Commun. 2020 Jan 21;11:402. doi: 10.1038/s41467-019-13960-2 (PMC6972792; doi:10.1038/s41467-019-13960-2)
Supplement: Supplementary file 7 — Source data [file 41467_2019_13960_MOESM7_ESM.zip › Source_Data/FigureS2/FigureS2B_MotifScanOutput/KmeansCluster1/homerResults/motif30.similar.html]

motif30

## Information for motif30

A
G
T
C
C
G
T
A
A
G
T
C
A
C
G
T
G
C
A
T
A
C
G
T
A
G
T
C
C
T
G
A
A
G
T
C
  
Reverse Opposite:  

A
C
T
G
A
G
C
T
A
C
T
G
G
T
C
A
C
G
T
A
C
G
T
A
A
C
T
G
A
C
G
T
A
C
T
G
  

|  |  |
| --- | --- |
| p-value: | 1e-27 |
| log p-value: | -6.279e+01 |
| Information Content per bp: | 1.922 |
| Number of Target Sequences with motif | 210.0 |
| Percentage of Target Sequences with motif | 4.12% |
| Number of Background Sequences with motif | 641.2 |
| Percentage of Background Sequences with motif | 1.62% |
| Average Position of motif in Targets | 94.3 +/- 66.5bp |
| Average Position of motif in Background | 103.4 +/- 67.3bp |
| Strand Bias (log2 ratio + to - strand density) | 0.3 |
| Multiplicity (# of sites on avg that occur together) | 1.02 |
| Motif File: | file (matrix) reverse opposite |

### Similar de novo motifs found

|  |  |  |  |  |  |  |  |
| --- | --- | --- | --- | --- | --- | --- | --- |
| Rank | Match Score | Redundant Motif | P-value | log P-value | % of Targets | % of Background | Motif file |
| 1 | 0.873 | A G T C C G T A G A T C A C G T A C G T C G A T T A G C C G T A T A G C G A T C C G T A A G T C | 1e-22 | -52.849811 | 0.75% | 0.04% | motif file (matrix) |
| 2 | 0.827 | A G T C C G T A A G T C A C G T A C G T G C A T T A G C C G T A T A G C G A T C C G T A A G T C A G C T | 1e-21 | -49.695639 | 0.63% | 0.03% | motif file (matrix) |
| 3 | 0.932 | A C T G A C G T A C T G C T G A C G T A C G T A A C T G A C G T T C A G A C T G | 1e-19 | -45.123075 | 1.67% | 0.44% | motif file (matrix) |
| 4 | 0.796 | C G T A A G T C A C G T A G C T C G A T A T G C C G T A A T G C G A T C C G T A A G T C | 1e-18 | -42.190682 | 1.00% | 0.16% | motif file (matrix) |
| 5 | 0.933 | C G T A A G T C A C G T G C A T A C G T A G T C C G T A A G T C | 1e-13 | -32.011840 | 4.73% | 2.68% | motif file (matrix) |
